# Evaluating the evidence for targeting FOXO3a in breast cancer: a systematic review

**DOI:** 10.1186/s12935-015-0156-6

**Published:** 2015-01-24

**Authors:** Simon Taylor, Matthew Lam, Chathyan Pararasa, James EP Brown, Amtul R Carmichael, Helen R Griffiths

**Affiliations:** Life and Health Sciences, Aston University, Aston Triangle, Birmingham, B4 7ET UK; Russells Hall Hospital, Dudley, DY1 2HQ UK

**Keywords:** Triple negative breast cancer, Phosphatidylinositol 3-kinase, Metabolism, Glycolysis, Oxidative stress, Apoptosis

## Abstract

**Background:**

Tumour cells show greater dependency on glycolysis so providing a sufficient and rapid energy supply for fast growth. In many breast cancers, estrogen, progesterone and epidermal growth factor receptor-positive cells proliferate in response to growth factors and growth factor antagonists are a mainstay of treatment. However, triple negative breast cancer (TNBC) cells lack receptor expression, are frequently more aggressive and are resistant to growth factor inhibition. Downstream of growth factor receptors, signal transduction proceeds via phosphatidylinositol 3-kinase (PI3k), Akt and FOXO3a inhibition, the latter being partly responsible for coordinated increases in glycolysis and apoptosis resistance. FOXO3a may be an attractive therapeutic target for TNBC. Therefore we have undertaken a systematic review of FOXO3a as a target for breast cancer therapeutics.

**Methods:**

Articles from NCBI were retrieved systematically when reporting primary data about FOXO3a expression in breast cancer cells after cytotoxic drug treatment.

**Results:**

Increased FOXO3a expression is common following cytotoxic drug treatment and is associated with apoptosis and cell cycle arrest. There is some evidence that metabolic enzyme expression is also altered and that this effect is also elicited in TNBC cells. FOXO3a expression serves as a positive prognostic marker, especially in estrogen (ER) receptor positive cells.

**Discussion:**

FOXO3a is upregulated by a number of receptor-dependent and -independent anti-cancer drugs and associates with apoptosis. The identification of microRNA that regulate FOXO3a directly suggest that it offers a tangible therapeutic target that merits wider evaluation.

## Background

Breast cancer is the third most frequent cancer worldwide. Amongst women, it is the most common malignancy, making up 21% of all new cancer diagnoses. Survival rates have been steadily extending over the past 50 years, primarily due to improvements in diagnosis and treatment.

The drivers of proliferation in breast cancer are also the phenotypic drug targets; hormone (estrogen, progesterone and HER2) receptors are commonly overexpressed. This knowledge has been harnessed in development of targeted therapies for breast cancer patients which inhibit hormone receptor activity and can be co-administered with the conventional, but non-specific, radiation and chemotherapy. The earliest approved therapy for endocrine related breast cancers was tamoxifen, an anti-estrogen receptor (ER)-targeting compound. These agents are commonly used alongside general chemotherapy that induces DNA damage through agents such as cisplatin which act to reduce the DNA damage repair response or anti-angiogenic agents [1,2].

The highly aggressive triple negative breast cancer (TNBC), with a prevalence of 15% of breast cancer cases often presenting in younger patients, is characterised by tumours that lack expression of ER, progesterone receptor and HER2/neu and is associated with a poor clinical prognosis [3]. There is no targeted treatment regime for this type of breast cancer and many patients experience relapse to cytotoxic chemotherapy within 3 years of diagnosis, as well as a higher incidence and probability of metastasis [4,5].

The forkhead box O (FOXO) family is activated by and limits the negative consequences of oxidative stress, metabolic dysregulation, growth factor withdrawal and DNA damage. At a molecular level, FOXO3a can be activated by an increased AMP/ATP ratio through phosphorylation catalysed by 5′ AMP-activated protein kinase (AMPK) at six conserved sites, five of which are located within the transactivation domain [6]. In response to a reduced energy supply FOXO3a transcription factors act as important regulators of cellular proliferation, cell cycle arrest, apoptosis, autophagy and metabolism [7,8].

Cancer cells may be distinguished from healthy cells in part by their metabolic phenotype. Solid tumours are often hypoxic and so express hypoxia-inducible factor 1 (HIF1), which increases expression of glycolytic enzymes, glucose transporters and inhibitors of mitochondrial metabolism allowing cellular adaptation through reliance on glycolysis to produce ATP in low oxygen environments [9,10]. This is associated with an increased cellular uptake of glucose in order to maintain energy homeostasis and is largely mediated upstream through PI3k-regulated FOXO transcription factors [11]. Removal of growth factors increases nuclear localisation of FOXO3a. Nuclear FOXO3a binds to P300, which then detaches from its transcription factor complex with HIF1 so suppresses glycolytic enzyme expression.

Phosphatidylinositol 3-kinase (PI3k)-activated Akt inhibits the activity of FOXO3a by phosphorylation of key residues, Thr32, Ser253 and Ser315; Akt-phosphorylated FOXO3a is then chaperoned by 14-3-3 proteins so obscuring FOXO3a-DNA binding sites and further preventing its activity within the nucleus [12]. FOXO3a is subsequently exported into the cytoplasm, ubiquitinated and degraded via the proteasome. Stress-induced phosphorylation of 14-3-3 by c-Jun N-terminal kinase (JNK) reduces 14-3-3 protein-FOXO3a binding capacity and directly inhibits nuclear export of FOXO3a thereby increasing transcriptional activity of FOXO3a [13]. Further metabolic control of FOXO3a activity is achieved following nuclear shuttling by acetylation and NAD^+^-dependent deacetylation.

**Figure 1:**
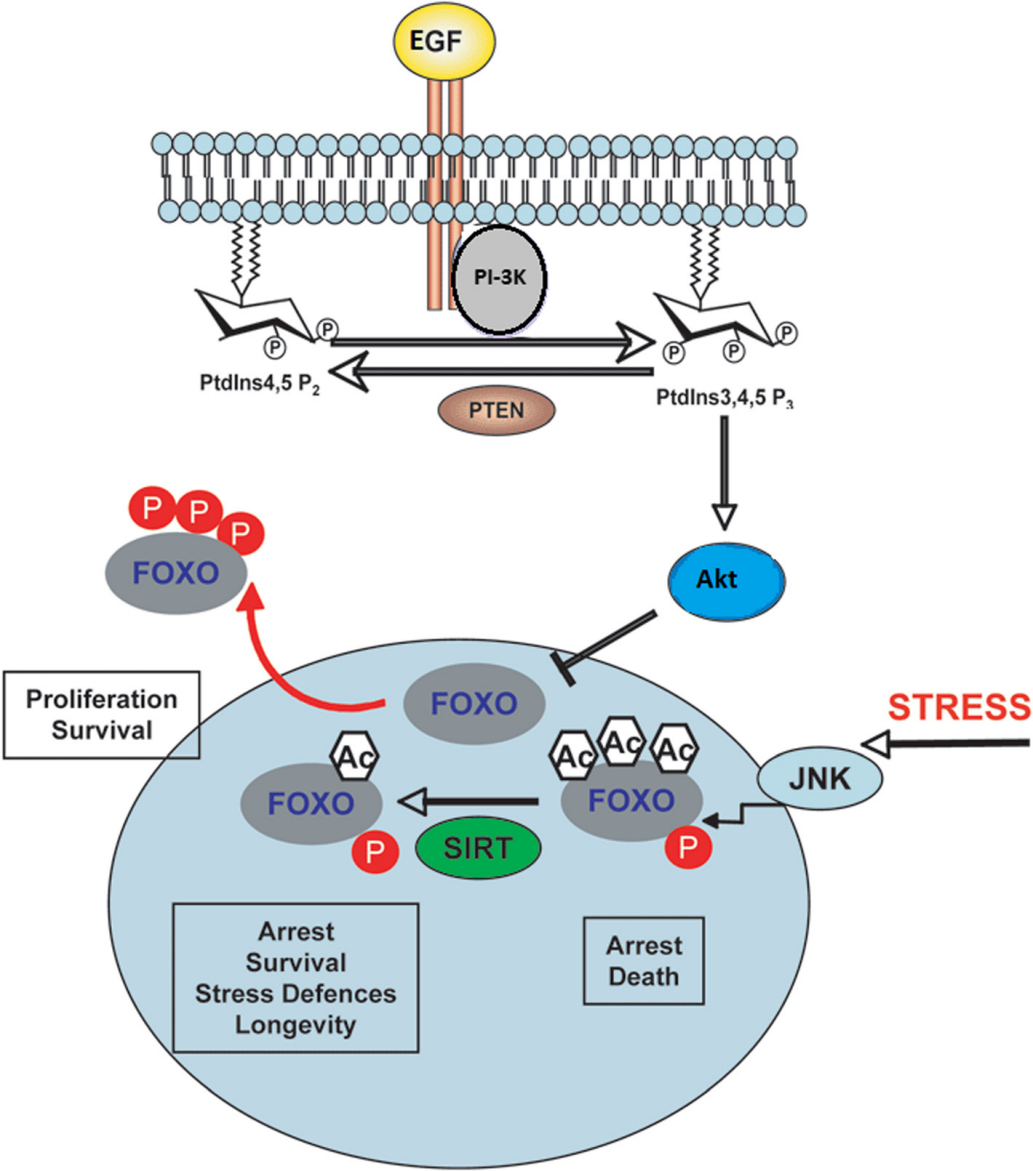
**FOXO3a regulation by Akt.** Growth factors/hormone stimulate PI3k phosphorylation of Akt, FOXO3a is phosphorylated by Akt, 14-3-3 also binds FOXO3a DNA binding sites, further preventing its activity in the nucleus. It is then tagged for degradation via ubiquitination and then degraded in a proteasome. Adapted from (Wilson, 2009 [49]).

Genomic analysis of TNBC has revealed frequent associations with mutations in class 1 PI3k which lies directly upstream of FOXO3a [14]. Mutations within the tumour suppressor PTEN, a negative regulator of Akt activation, can result in constitutively active Akt and inhibit FOXO3a, so promoting HIF1 expression, antioxidant gene expression and an increase membrane translocation of glucose transporters and rate-limiting enzymes such as phosphofructokinase-1 [15] (Figure 1).

Down-regulation of FOXO3a activity is often seen in cancers and ERK- or inhibitor κappa B kinase (IκKβ)-mediated inhibition of FOXO3a has been shown to promote tumorigenesis [16,17]. Despite evidence for FOXO3a down-regulation in breast cancer, the AMPK-FOXO3a pathway is still inducible, providing a potential therapeutic target for cancer chemotherapy which is independent of receptor status [6]. This evidence highlights FOXO3a as a potentially important target by cytotoxic drugs especially in receptor-negative cells. To investigate the validity of this hypothesis, we have undertaken a systematic approach to analysis of the published literature which describes the effects of breast cancer chemotherapeutic agents on FOXO3a. The goal is to determine whether FOXO3a represents a critical target of therapeutics used in the treatment of breast cancer, and therefore whether the evidence supports direct targeting of FOXO3a in chemoresistant-resistant disease notably in TNBC.

## Results

**Figure 2:**
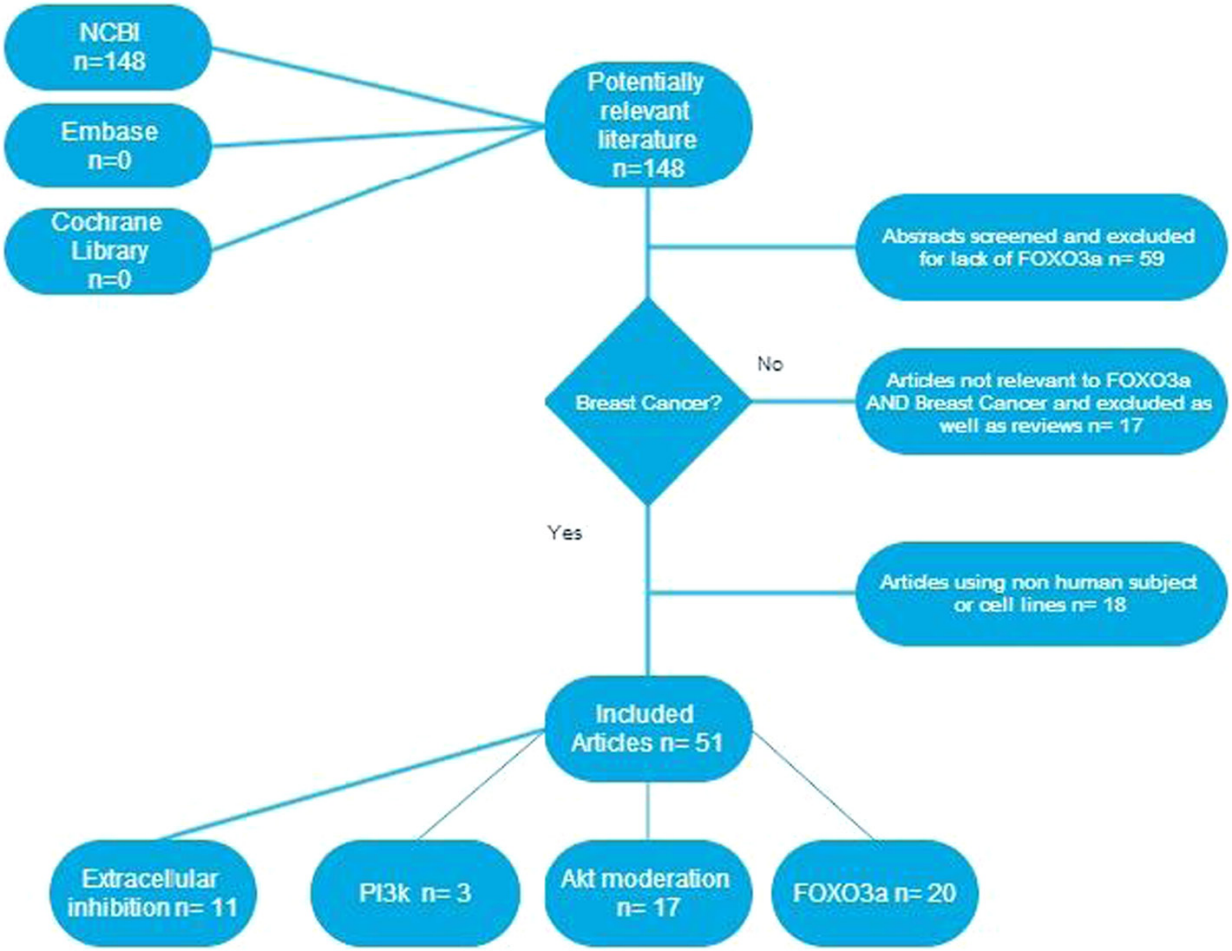
**Flowchart showing the retrieval and review of literature according to systematic criteria.**

The systematic search for relevant articles initially retrieved 148 articles. The 51 articles that met the inclusion criteria were then analysed and categorised according to the drug target (extracellular receptor; PI3k; AKT; FOXO3a), and assessed for dependency on FOXO3a activity. Twenty articles met these conditions and have been analysed here (Figure 2).

Activation of growth factor receptors signals to promote tumour progression, cell survival and invasive characteristics. ER-positive cells are reported to have higher FOXO3a levels and increased apoptotic activity with less invasive characteristics than ER-negative cells. The ER status of breast tumours is an important indicator of prognosis, and although ER signaling plays a major role in tumour progression, ER-positive cancer is also associated with better prognosis than ER-negative breast cancers [18].

**Figure 3:**
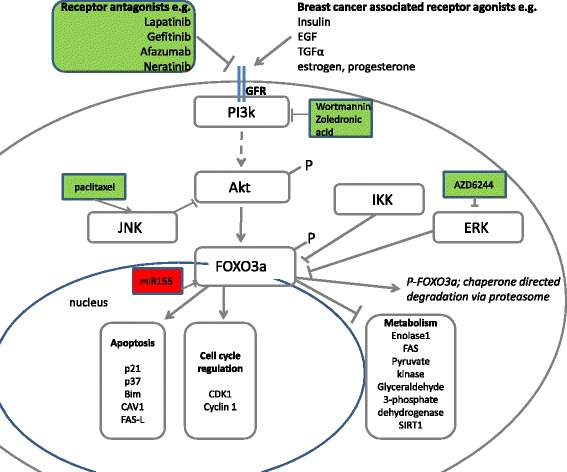
**Schematic illustration of mechanisms of action of chemotherapeutic agents used in breast cancer therapy upstream of FOXO3a expression.** Chemotherapeutic agents in green boxes increase FOXO3a expression/activity; those in red boxes decrease FOXO3a expression/activity. CAV1 = caveolin 1, CDK1 = cyclin dependent kinase 1, ERK = Extracellular signal-regulated kinases, FAS-L = FAS ligand, FAS = fatty acid synthase, FOXO3a – forkhead box O3a, GFR – growth factor receptor IκKβ – inhibitor kappa kinase beta, JNK - Jun N-terminal kinases, PI3k - phosphatidylinositol 3-kinase, SIRT1 = sirtuin 1.

Following a systematic review of the literature, five articles were retrieved which described the effects of growth factor receptor inhibition on FOXO3a activity (Table 1). The mechanisms of action of anti-cancer agent classes upstream from FOXO3a are illustrated in Figure 3.

**Table 1:** **Growth factor receptor antagonists consistently activated FOXO3a in sensitive breast cancer cells**

**First author (Year)**	**Treatment**	**Effect on FOXO3a (Activates/Inactivates)**	**Cellular effects**
**Hegde (2007)** [22]	Lapatinib	Activates in responsive cell lines; measured as Thr^32^ P-FOXO3a and by microarray, BT474 and SKBr3. No effect in resistant cell lines; MDA-MB-468 and T47D.	Decreased expression of glyceraldehyde-3-phosphate dehydrogenase, enolase 1, pyruvate kinase and fatty acid synthase expression in BT474 and SKBr3
			Growth rate reduced in BT474 and SKBr3
**Karadedou (2012)** [23]	Lapatinib	Activates in BT474 or SKBR3 measured by FOXO3a nuclear translocation.	Decreased expression of VEGF.
**Real PJ (2005)** [20]	Trastuzumab	Activates in MB231 and SUM159, primary breast effusions; measured as nuclear translocation of FOXO3a.	Up-regulation of Bnip1.
			Increased sensitivity to apoptosis.
**Krol (2007)** [19]	Gefitinib	Activates in BT474 and SKBR3, but no effect in gefitinib-resistant lines MCF-7, MDA-MB-231, and MDA-MB-453. Measured as Thr^32^ P-FOXO3a and nuclear translocation analysis.	Cell cycle arrest predominantly at the G(0)-G(1) phase and apoptosis.
**McGovern (2009)** [21]	Gefitinib	Activates in BT474 and SKBR3 but not the gefitinib-resistant lines MCF-7, MDA-MB-231, and MDA-MB-453. Measured as nuclear FOXO3a and microarray.	Increase in Bim, p27 ^kip1^

Irrespective of the target receptor for each of the inhibitors, lapatinib and trastuzumab (epidermal growth factor receptor; EGFR and/or EGFR2; HER2, respectively), or whether it targeted receptor-associated tyrosine kinase (gefitinib), FOXO3a was consistently activated and translocated to the nucleus. Three studies confirmed an increase in apoptotic gene expression and extent of apoptosis [19-21] after anti-growth factor receptor antibody treatment. Only one study reported effects on metabolic gene expression with a switch away from anabolic metabolism [22] while Karadedou reported the inhibition of angiogenic VEGF expression [23].

Downstream from ligand-receptor binding, receptor-associated PI3k is activated and catalyses the production of phosphatidyl inositol (PtdIns) 4,5 bisphosphate and PtdIns 3,4,5 trisphosphate that act as second messengers, and via PDK1 activation results in Akt phosphorylation. The importance of PI3k for signal transduction is evidenced by the work of Reagan-Shaw *et al.* who showed activation of FOXO3a and induction of apoptosis when PI3k is knocked down [24]. The bisphosphonate, zoledronic acid, originally used for osteoporosis management, is now in clinical trials as a chemotherapeutic drug; it activates FOXO3a and inhibits expression of the proangiogenic factor CCN1 (Table 2) [25]. Zoledronic acid when used as an adjuvant to endocrine therapy in premenopausal women with hormone receptor-positive early breast cancer provides clinical benefit and is cost-effective [26].

**Table 2:** **PI3k inhibition causes FOXO3a activation in breast cancer cells**

**First author (Year)**	**Treatment**	**Effect on FOXO3a (Activates/Inactivates)**	**Cellular effects**
**Espinoza (2011)** [25]	Zoledronic acid (ZOL)	Activates in MDA-MB-231 and MCF-7 measured by nuclear translocation of FOXO3a.	Inhibited proangiogenic factor, CCN1 in TNBC
**Guo (2004)** [34]	Wortmannin, EGCG	Activates in MCF-7 and ZR-75 cells; and Hs578T and MDA-MB-231 cells, measured as FOXO3a expression and nuclear translocation.	Increased ER expression

The phosphorylation of Akt indirectly via PDK1 activation by PI3k increases Akt activity which, in turn, phosphorylates FOXO3a. A number of small molecule inhibitors of Akt were identified in the review as consistently activating FOXO3a with subsequent arrest of the cell cycle (via p21 ^cip1^ and p27 ^kip1^ expression, [27,28]) and induction of apoptosis (Bim, [29-33]); Table 3. The majority of the small molecules did not target AKT directly but mediated AKT activation via unknown targets and other kinases such as JNK and P38. Effects on expression of ER differed between inhibitors treatments [27,34].

**Table 3:** **AKT inhibition activates FOXO3a in breast cancer cells**

**First author (Year)**	**Treatment**	**Effect on FOXO3a (Activates/Inactivates)**	**Cellular effects**
**Brandi (2013)** [27]	Indole-3-carbinol cyclic tri- and tetrameric derivatives, specific target unknown but inhibits AKT directly or indirectly.	Activates in MCF-7 and MDA-MB-231 breast cancer cell lines) and in vivo in a tumour xenograft measured as nuclear translocation of FOXO3a.	Increased expression of p21 ^cip1^, p27 ^kip1^ and decreased ER expression.
**Li (2007)** [29]	Selenium and Doxorubicin via p38 mediated inhibition of AKT.	Activates in MCF7 measured by P-FOXO3a and reporter assay.	Increased Bim expression and apoptosis.
**Sharma (2012)** [33]	18β-glycyrrhetinic acid (GRA) specific target unknown but inhibits AKT directly or indirectly.	Activates in MCF7 but not normal breast cell line MCF-10 measured as increased expression and nuclear translocation.	Increased Bim expression and caspase-dependent apoptosis.
**Sunters (2006)** [32]	Paclitaxel inhibits AKT via JNK	Activates in MCF7 measured as nuclear localisation of FOXO3a.	JNK1 activation and apoptosis in MCF7 and also in a panel of other cells lines MT 3522, 734 B, ZR-75-1, T47-D, CAL-51, CAMA-1, MDA-MB-231, and SKBR-7.
**Xie (2010)** [31]	SZ-685C (marine anthraquinone) specific target unknown. Inhibits AKT directly or indirectly.	Activates in MCF-7 and MDA-MB-435.	AKT inhibition.
			Increased Bim.
			Increased apoptosis.
			Increased caspase activity.
**Zhao (2013)** [30]	5,7-dihydroxy-8-nitrochrysin (NOC)-specific target unknown. Inhibits AKT directly or indirectly.	Activates in MDA-MB-453.	Increased Bim expression
			Increased apoptosis.
**Lin (2011)** [28]	FLOT1 silencing associated with suppression of Akt activity	Activates in MCF7 and MDA-231 measured as expression level and P-FOXO3a.	Up-regulation of p21 ^cip1^ and p27 ^kip1^

A number of regulatory (patho)-physiological microRNA (miR) and small molecule activators have been shown to target FOXO3a directly and regulate its nuclear localisation and transcriptional activity (Table 4; [35-40]).

**Table 4:** **FOXO3a activation in breast cancer cells increases apoptosis**

**First author (Year)**	**Treatment**	**Effect on FOXO3a (Activates/Inactivates)**	**Cellular effects**
**Kong (2010)** [35]	miR-155	Inhibits in BT-474 measured by protein expression.	Decreased Bim and p27 expression decreased apoptosis.
**Kong (2012)** [36]	AZD6244, indirectly as an ATP-uncompetitive inhibitor of MEK ½	Activates FOXO3a in MTDH knock-down, AZD6244 resistant lines.	Increased apoptosis.
**Lam (2012)** [37]	Aqueous extract of Fagonia	Activates FOXO3a measured by Western blot in MCF7 and MDA231.	Cell cycle arrest and apoptosis.
**Lin (2010)** [38]	miR-96	Inhibits in BT549, ZR-75-30, Bcap37, MDA-MB231, MDA-MB435, MCF-7, SKBR3 measured as FOXO3a expression and by reporter assay.	Down-regulation of p21 ^cip1^, p27 ^kip1^, CDK and cyclin 1.
**Liu (2012)** [39]	Arsenic trioxide	Activates in MCF7 measured as nuclear translocation and expression.	Decreased IKKB.
			Increased apoptosis.
**Stan (2008)** [40]	Withaferin.	Activates in MCF-7 (estrogen-responsive) and MDA-MB-231.	Increased Bim expression.
			Increased apoptosis.

miR155, which is up-regulated in cancer, is a negative regulator of FOXO3a and subsequently Bim and p27 ^kip1^ expression, but inhibition of miR155 restores sensitivity to apoptosis [35]. Similarly, miR96 suppressed expression through the 3′-UTR on the FOXO3a gene, results in down-regulation of p21^cip1^, p27^kip1^ and cyclin dependent kinases [38]. Modulation of FOXO3a by anti-miR may prove useful to promote apoptosis. Other small molecules appear to modulate FOXO3a activity by regulating the activity of FOX3a-regulating kinases such as IκKβ and MEK so preventing FOXO3a phosphorylation, increasing its nuclear half-life and transcriptional activity.

## Discussion

There is a growing need for new drug targets in cancer treatment. These should efficiently combine fewer side-effects with lower likelihood of resistance development. The multi-step growth factor, PI3k/PTEN, Akt and FOXO3a pathway provides a potentially important target network. Dysregulation of its components, such as PTEN, PIK3CA, and AKT (PKB), are common in solid tumours but despite evidence for FOXO3a down-regulation in breast cancer, the AMPK-FOXO3a pathway is still inducible.

This systematic review has highlighted that several different classes of drugs can increase FOXO3a activation and promote apoptosis by targeting many of the key steps in the pathway downstream of the receptor in breast cancer cell lines irrespective of hormone receptor status. There is some evidence that metabolic pathways are also affected when FOXO3a is targeted, favouring oxidative phosphorylation rather than glycolysis. Our systematic literature approach has largely retrieved papers published around FOXO3a effects on cell cycle control and induction of apoptosis. The importance of metabolic switching as a mechanism for FOXO3a up-regulation in slowing growth of breast cancer cells has been reported in one study only, probably because the published studies have limited their focus to analysis of death and death pathways.

Recently, a dual network strategy for therapeutic intervention has been proposed to target various diseases [41]; such an approach inhibits central nodes in disease and also their influencing networks based on the premise that during disease progression the importance of any given target may change and so may be best approached using sequential multi-target therapy [41]. If FOXO3a expression changes occur early in the lifecycle of breast cancer, the metabolic switch through a FOXO3a node may prove to be an important route to delaying disease progression.

FOXO3a can inhibit expression of glycolytic enzymes through HIF-1 inhibition and can regulate mitochondrial gene expression, ACO2, LARS2, MRPLI2, OXNAP1 and ATP5G1, which are typically down-regulated in hypoxia [42]. The combined effect is to starve hypoxic tumours and prevent further growth ultimately leading to autophagy and death.

FOXO3a expression is a positive prognostic marker for breast cancer [43]. The activity of FOXO3a, which regulates ERα expression, is likely to be responsible. The expression of ERα is associated with a higher degree of differentiation of tumours and lower speed of tumour cell proliferation [44], it can activate the cell cycle progression through either genomic or non-genomic pathways [45], and estrogen-inducible genes can suppress tumour progression [46]. However, ER knockdown results in a switch towards increased invasiveness in the presence of increased FOXO3a expression suggesting that the nuclear receptor represents a crucial switch in FOXO3a control of breast cancer cell aggressiveness [18]. Stratification of patients according to receptor positivity may be critical to improve the benefit:risk ratio of targeting FOXO3a.

Low FOXO3a expression is associated with poor prognosis in a number of other cancers including neuroblastoma, gastric adrenocarcinoma, hepatocarcinoma and poor metastasis-free survival in renal cell carcinoma [47-50]. In common with observations in breast cancer cells, treatment of colon cancer cells with selenite induced ROS-dependent, FOXO3a-mediated apoptosis [51]. Enhancing FOXO3a expression appears to be a relevant to treatment for a number of cancers. One emerging means of modulating FOXO3a expression therapeutically is post-transcriptionally via specifically targeted miR. In this review, we have identified two papers that report the effects of specific miR which affect FOXO3a expression in breast cancer cells. It has been estimated that there may be thousands of target genes for miR-155, although significant overlap has been observed between miR-155 targets and the molecular profile of mutant p53-expressing breast tumours suggesting that this may prove to be a particularly useful target with fewer side-effects [52]. Far fewer genes have been has been described as targets for miR-96 although existing works share the common conclusion that interference with miR-96 using antisense miRNA (antagomiR) molecules increase apoptosis in breast cancer cells. More work is needed before to understand potential off-target effects before their potential as therapeutic targets for breast cancer can be appreciated [53].

Chemotherapeutic drug resistance can limit the application of many anti-cancer therapies. Acquired resistance to lapatinib and trastuzumab frequently occurs in breast cancer patients, possibly as a consequence of FOXO3a de-repression and increased ER signaling [54], however, this does present an opportunity for adjuvant therapy with drugs such as tamoxifen that target ER. Similarly, upregulation of HER3 is induced in a FOXO3a dependent way which attenuates the beneficial effects of PI3k inhibitors unless used in combination with HER2/3 antagonists [55]. Indeed preclinical data support the suggestion that targeting of the PI3k/mTOR pathway in combination with trastuzumab is beneficial in trastuzumab-resistant breast cancer [56]. The studies highlighted here show the potential for targeting the upregulation of FOXO3a to overcome resistance to AZD6244 [35].

The clinical trials register lists two studies currently underway which will assess FOXO3a status (albeit not as primary endpoints) one using an ER antagonist with a pan-class I PI3k inhibitor and the other using reparaxin, an IL-8 receptor antagonist.

## Conclusion

The identification of new classes of FOXO3a inhibitors offers a promising strategy for future anti-cancer drug design by targeting a downstream node of the PI3k pathway that is not commonly mutated in cancers. In the age of personalised medicine and following the identification of regulatory miR that target FOXO3a directly, their inhibition as an adjunct therapy alongside conventional cytotoxic and/or receptor antagonists in stratified patient groups merits evaluation.

## Methods

We have designed a systematic literature review to identify published studies describing the effects of anti-cancer drugs on FOXO3a in: human cell lines or primary cells from breast cancer patients and from immunohistochemical analyses of tissue.

PubMed/Medline, Cochrane and Embase databases were searched from 1 January 2014 to 1 April 2014. Neither the Cochrane library nor Embase yielded relevant results using specific search criteria. Therefore PubMed/Medline was the database used for information, the Boolean search terms are listed below: ((FOXO3a[All Fields] OR (Forkhead[All Fields] AND Box[All Fields] AND O3[All Fields])) OR (Forkhead[All Fields] AND Box[All Fields] AND (“proteins”[MeSH Terms] OR “proteins”[All Fields] OR “protein”[All Fields]) AND 3[All Fields])) AND (“breast neoplasms”[MeSH Terms] OR (“breast”[All Fields] AND “neoplasms”[All Fields]) OR “breast neoplasms”[All Fields] OR (“breast”[All Fields] AND “cancer”[All Fields]) OR “breast cancer”[All Fields]).

Results were limited to peer-reviewed, English language articles only. Reviews, meta-analyses, case reports, editorials and letters lacking primary data were excluded.

## Abbreviations

AMPK: AMP-activated protein kinase
ER: Estrogen receptor
EGFR: Epidermal growth factor receptor
HER2: Human epidermal growth factor like 2
FOXO3a: Forkhead box O3a
HIF1α: Hypoxia inducible factor 1alpha
IκKβ: Inhibitor kappa kinase beta
JNK: Jun N-terminal kinases
PI3k: Phosphatidylinositol 3-kinase
MiR: microRNA
TNBC: Triple negative breast cancer
